# DBSCAN-PCA-INFORMER-Based Droplet Motion Time Prediction Model for Digital Microfluidic Systems

**DOI:** 10.3390/mi16050594

**Published:** 2025-05-19

**Authors:** Zhijie Luo, Bin Zhao, Wenjin Liu, Jianhua Zheng, Wenwen Chen

**Affiliations:** 1 College of Information Science and Technology, Zhongkai University of Agriculture and Engineering, Guangzhou 510225, China; jackeylzj@163.com (Z.L.);; 2 Intelligent Agriculture Engineering Technology Research Centre, Zhongkai University of Agriculture and Engineering, Guangzhou 510225, China; 3 Guangzhou Key Laboratory of Agricultural Products Quality & Safety Traceability Information Technology, Zhongkai University of Agriculture and Engineering, Guangzhou 510225, China

**Keywords:** digital microfluidics, microdroplets, DBSCAN-PCA-INFORMER model, time prediction

## Abstract

In recent years, emerging digital microfluidic technology has shown great application potential in fields such as biology and medicine due to its simple structure, sample-saving properties, ease of integration, and wide range of manipulation. Currently, due to potential faults in chips during production and usage, as well as high safety requirements in their application domains, thorough testing of chips is essential. This study records data using a machine vision-based digital microfluidic driving control system. As chip usage frequency rises, device degradation introduces seasonal and trend patterns in droplet motion time data, complicating predictive modeling. This paper first employs the density-based spatial clustering of applications with noise (DBSCAN) clustering algorithm to analyze the droplet motion time data in digital microfluidic systems. Subsequently, principal component analysis (PCA) is applied for dimensionality reduction on the clustered data. Using the INFORMER model, we predict changes in droplet motion time and conduct correlation analysis, comparing results with traditional long short-term memory (LSTM), frequency-enhanced decomposed transformer (FEDformer), inverted transformer (iTransformer), INFORMER, and DBSCAN-INFORMER prediction models. Experimental results show that the DBSCAN-PCA-INFORMER model substantially outperforms LSTM and other benchmark models in prediction accuracy. It achieves an R^2^ of 0.9864, an MSE of 3.1925, and an MAE of 1.3661, indicating an excellent fit between predicted and observed values.The results demonstrate that the DBSCAN-PCA-INFORMER model achieves higher prediction accuracy than traditional LSTM and other approaches, effectively identifying the health status of experimental devices and accurately predicting failure times, underscoring its efficacy and superiority.

## 1. Introduction

Digital microfluidic chips have recently emerged as a novel technology, offering several advantages over traditional continuous microfluidic chips. Firstly, digital microfluidic chips can significantly reduce sample consumption, effectively minimizing resource waste while simplifying sample processing workflows and enhancing operational convenience and time efficiency. Secondly, unlike continuous microfluidic chips, digital microfluidic chips do not require the design of microchannel structures, which reduces the risk of sample contamination and eliminates dead zones, thereby improving experimental accuracy. From a technical perspective, the structure of digital microfluidic chips is relatively simple, omitting the need for components like micro-pumps, making integration easier and lowering fabrication costs. This technology has a wide range of applications, enabling precise manipulation of various samples [1], including but not limited to gene sequencing, cell analysis, and drug development [2,3,4]. These advantages position digital microfluidic chips as a forefront technology in the field of microfluidics [5,6]. However, as application scenarios become more diverse and usage frequency increases, digital microfluidic devices still face multiple challenges: electrode aging and failure risks under high-voltage driving, droplet “sticking” on electrode surfaces causing transport faults, and the slow response of traditional monitoring methods to sudden anomalies. These issues severely limit the deployment of digital microfluidic technology in scenarios requiring high reliability. Over the past decade, digital microfluidics has rapidly developed and become a focal point of international research [7,8,9,10]. To date, most studies have focused on device design and functional implementation, and there is a lack of real-time prediction and fault-warning methods for the health status of devices. In particular, systematic research on droplet motion time—a key performance indicator—essentially remains non-existent. However, digital microfluidic devices rely on high-voltage drivers and frequently encounter defects, with droplets often “sticking” to certain drive electrodes. Thus, improving the reliability of digital microfluidic devices is essential. This study proposes a prediction model for droplet motion time in digital microfluidic chips, aiming to provide a basis for algorithm planning of continuous droplet transport paths, thereby mitigating the impact of drive electrode failures on various droplet applications.

Artificial neural networks have advanced rapidly in recent years, demonstrating effectiveness in function approximation and data prediction. However, a review of the literature reveals a lack of studies specifically focused on droplet motion time prediction in digital microfluidic systems. Recurrent neural networks (RNNs) are capable of learning intricate patterns in time series data but face challenges like gradient vanishing and explosion. Long short-term memory (LSTM) networks address these issues by incorporating gating and memory units, allowing for selective retention of useful data, thus effectively handling long-term time series data. Several studies have utilized LSTM for time series prediction, such as Wang et al. [11], who proposed an LSTM-based strategy for energy consumption forecasting and Huang et al. [12]. Luo et al. [13] proposed a precipitation nowcasting system based on LSTM networks to predict future precipitation intensity. Convolutional neural networks (CNNs) [14,15,16,17] are also helpful in extracting valuable features and filtering noise from input data. However, as the use of digital microfluidic chips increases, device degradation intensifies, and the seasonal and trend patterns in droplet motion time data complicate predictions, thereby reducing accuracy.

Recent literature [18] has introduced the encoder–decoder transformer model, which does not require consideration of time and distance in time series predictions, offering potential for handling long-term dependencies and parallel computation and significantly reducing computational resource consumption. The INFORMER model was developed to reduce the time complexity of transformer models by optimizing the self-attention mechanism with sparse probability self-attention. This innovation lowers time complexity and enables long-sequence outputs with a single fully connected layer.

In this work, we propose a DBSCAN-PCA-INFORMER neural network model that integrates a preprocessing pipeline combining density-based clustering (DBSCAN) and principal component analysis (PCA) with an enhanced transformer model—INFORMER. By leveraging sparse probabilistic self-attention, the model efficiently captures long-sequence dependencies to achieve highly accurate predictions of droplet motion time. This method not only filters out anomalous noise but also provides real-time feedback on device operating status, offering crucial data for subsequent fault localization and path-planning optimization. To evaluate its effectiveness, the model is benchmarked against conventional deep learning approaches including long short-term memory (LSTM), frequency-enhanced detrcomposed ansformer (FEDformer), inverted transformer (iTransformer), INFORMER, and DBSCAN-INFORMER. The experimental results demonstrate that the DBSCAN-PCA-INFORMER model excels in error prediction and can accurately infer device status and the extent of drive electrode failures. This provides strong support for the scientific assessment of device health and helps minimize the impact of drive electrode failures on droplet applications, thereby laying a solid foundation for future research and practical applications in the field of digital microfluidics.

## 2. Mechanism of Digital Microfluidic Systems Based on Dielectrophoresis

**Figure 1:**
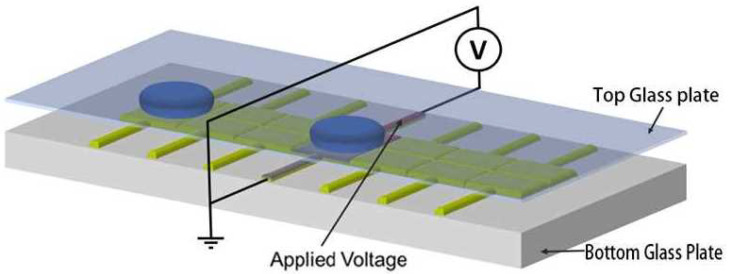
Illustration of a digital microfluidic chip.

At present, there are two primary types of chip structures for digital microfluidic devices based on dielectrophoresis: closed and open structures. In open chip structures, droplets are directly placed on a single substrate that contains both driving and ground electrodes. This design has several advantages, including low friction and a simple fabrication process. However, droplets in open systems are prone to evaporation and can be affected by external environmental factors, making it difficult to achieve complex droplet manipulations and applications. As a result, closed chip structures are increasingly used. Figure 1 shows the structure of a bipolar plate closed digital microfluidic system chip. In this configuration, the upper substrate serves as a ground electrode made of a continuous layer of transparent conductive indium tin oxide (ITO), while the lower substrate features an array of driving electrodes that have been etched using a series of photolithography processes. The driving electrodes are typically square in shape. Both substrates are covered with a dielectric layer and a hydrophobic layer. The dielectric layer primarily enhances the reliability of the device and reduces the likelihood of electrical breakdown due to voltage. Once the chip is assembled, droplets are injected into the space between the upper and lower substrates for operation. Generally, droplets operate in air; however, more researchers are using materials such as silicone oil, which do not mix with the droplets, as filling media to reduce the driving voltage required for droplet manipulation. It is important to note that while the addition of silicone oil can reduce the resistance to droplet movement, its introduction may potentially impact the application of chemical synthesis experiments on microfluidic chips. Therefore, the filling medium in bipolar plate closed chip structures needs to be determined based on the application environment.

## 3. DBSCAN Dimensionality Reduction Algorithm

Due to the fluctuations in the droplet movement time data of digital microfluidic chips, the dataset may contain a certain scale of outliers, resulting in a non-spherical shape distribution, which is not conducive to clustering using the k-means algorithm. However, the DBSCAN (density-based spatial clustering of applications with noise) algorithm, which is density based, can avoid the influence of outliers and is suitable for clusters of any shape [19]. k-means requires specifying the number of clusters in advance, while DBSCAN does not require manual specification, instead needed setting of the neighborhood radius (Eps) and the minimum number of points (MinPts). The DBSCAN algorithm automatically clusters the data by setting two key parameters—the neighborhood radius (Eps) and the minimum number of points (MinPts)—while also filtering out outliers. In terms of parameter settings, the proposer of the DBSCAN algorithm sets MinPts to 4, and generally, for two-dimensional clustering, it is also set to 4. For Eps, it can be determined using the k-distance method with the following steps:

(1) Given a *k* value, typically taken as k=2∗dim−1, where dim is the dimension of the input data.
(2) For every data point, calculate the distance to its k-nearest neighbors and sort them in descending order.
(3) Find the abrupt points (inflection points) in the sequence from step (2), which will determine the value of Eps.

To illustrate the algorithm, the basic concepts in DBSCAN are provided:

(1) Neighborhood: The region around a given object with a radius of Eps.
(2) Core Point: A point within the neighborhood radius Eps that has a number of data points greater than or equal to MinPts.
(3) Border Point: A point within the neighborhood radius Eps that contains fewer than MinPts data points but lies within the neighborhood of a core point.
(4) Noise Point: A point that is not a core point or a border point.
(5) Density Reachable: Other points *Q* located in the vicinity of core point *P* are described as density reachable from *P*.
(6) Density Reachability: Given a sample set *H*, where *n* sample points are $h_{1},h_{2},\ldots ,h_{i},\ldots ,h_{n}$, and $p=h_{1}$ and $q=h_{n}$. if $h_{i}$ is density reachable to $h_{i+1}$, then *p* is said to be density reachable to *q*.
(7) Density Connected: In the same dataset *H*, if h1 is density reachable from $h_{2}$ and $h_{3}$, then $h_{2}$ and $h_{3}$ are density connected.

The basic idea of the DBSCAN algorithm for determining clusters is to mark all data points in the sample as unvisited. A random unmarked point *k* is selected to start clustering. If the number of data points in the neighborhood of *k* is greater than or equal to MinPts, then point is marked as a core point, and all density reachable points are found and clustered together. This process continues until all data points are visited. Finally, data points that fail to be grouped into any cluster are identified as noise points or outliers.

## 4. The Basic Principles of the INFORMER Neural Model

The INFORMER model is an innovative transformer architecture built upon the attention mechanism, designed to address the issues encountered by traditional transformer models in time series forecasting, including the quadratic time complexity associated with the self-attention mechanism, memory bottlenecks caused by stacked layers, and slow prediction speed [20]. To emphasize dominant attention while reducing output dimensions and network parameters, a self-attention distillation mechanism is proposed. This mechanism applies regular convolutions and pooling operations to the output of each attention layer, halving the output of each layer, allowing the model to focus more on key information while alleviating the computational burden of the network. A parallel generative decoder mechanism is introduced, enabling all prediction results to be output in a single forward computation, avoiding error accumulation and improving the speed of inference in time series forecasting.

**Figure 2:**
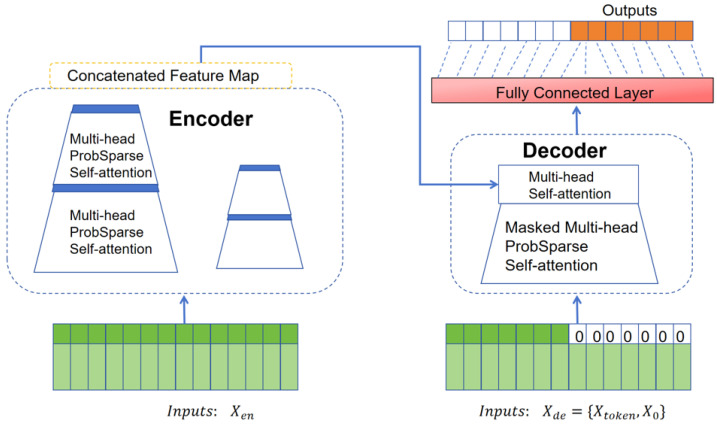
INFORMER model.

Figure 2 illustrates the overall architecture of the INFORMER model. The encoder extracts dependencies between input variables, while the decoder generates the predictions. On the left side of Figure 2, the encoder processes the long-sequence input through the probabilistic sparse self-attention module and the self-attention distillation module, capturing feature representations and dependencies among input variables. The right side shows the decoder, where multi-head attention interacts with the encoded features from the long sequence data and the placeholder ”0” for the predicted target part to output the prediction data.

### 4.1. Probabilistic Sparse Self-Attention Mechanism

The probabilistic sparse self-attention mechanism in the INFORMER model is an approach to sparsify the self-attention matrix from a probabilistic angle, lowering computational complexity to logarithmic linear levels. The self-attention mechanism in the transformer takes three input matrices—query vectors, key vectors, and value vectors—and calculates a weighted sum of the value vectors.

(1) $$\begin{array}{cc} A(Q,K,V) & =\mathrm{Softmax}\left(\frac{Q^{T}}{\sqrt{d}}\right)V\\ Q & \in R^{L_{Q}\times d},K\in R^{L_{K}\times d},V\in R^{L_{v}\times d} \end{array}$$

The notation indicates the set of real numbers and represents the input dimension.

The probabilistic form of the attention coefficient for the *i*-th query vector is

(2) $$A\left(q_{i},K,V\right)=\sum_{j}\frac{k\left(q_{i},k_{j}\right)}{\sum_{l}\left(q_{i},k_{j}\right)}v_{j}=E_{p\left(k_{j}|q_{i}\right)}\left[V_{j}\right]$$

In the expression, $k\left(q_{i},k_{j}\right)$ represents the asymmetric exponential kernel function $\exp\left(\frac{q_{i},k_{j}^{T}}{\sqrt{d}}\right)$, *E* represents the expected value, *p* represents the conditional probability, $q_{i}$ represents the *i*-th row of the *Q* matrix, and $k_{j}$ and $v_{j}$ represent the *j*-th row of the *K* and *V* matrices, respectively.

In the attention mechanism of the transformer, there is a sparsity problem, which manifests as a long-tailed distribution phenomenon in the self-attention feature map [15]. A small number of dot products contribute the majority of the attention scores, and most pairwise dot product calculations can be ignored. To measure the sparsity of the query vector, Kullback–Leibler divergence (KL divergence) is used to compute the relative entropy between the probability distribution of the query vector and the uniform distribution. The evaluation formula for the sparsity of the *i*-th query vector is as follows:

(3) $$M\left(q_{i},\;K\right)=\ln\sum_{j=1}^{L_{K}}\frac{q_{i}k_{j}^{T}}{e^{\sqrt{d}}}-\frac{1}{\;L_{K}}\sum_{j=1}^{L_{K}}\frac{q_{i}k_{j}^{T}}{\sqrt{\;T}}$$

In this formula, the first term represents the log-sum-exp (LSE) computation across all key vectors, while the second term denotes the arithmetic average. Memory usage remains $O\left(L_{Q}L_{K}\right)$, and there are numerical stability issues with LSE. By evaluating the sparsity of the query vector, an approximation is obtained as follows:

(4) $$\bar{M}\left(q_{i},\;K\right)=\max_{j}\left\{\frac{q_{i}k_{j}^{T}}{\sqrt{\;d}}\right\}-\frac{1}{\;L_{K}}\sum_{j=1}^{L_{K}}\frac{q_{i}k_{j}^{T}}{\sqrt{\;d}}$$

By randomly selecting $u\left(u=L_{Q}\ln L_{K}\right)$ dot product operations $\bar{M}\left(q_{i},K\right)$, the complexity is reduced to O(LlnL). The largest *u* query vectors are then chosen, and the formula for ProbSparse self-attention is obtained as follows:

(5) $$A\left(Q,K,V\right)=\mathrm{Softmax}\left(\frac{\bar{Q}K^{T}}{\sqrt{\;d}}\right)V$$

In the formula, $\bar{Q}$ represents the filtered query vectors, which include only the top *u* key query vectors.

The U query vectors with the highest scores are selected, and the attention scores for each key value are calculated based on the top key query vectors. The remaining query vectors are set to the average value of the inputs to the self-attention layer. By focusing on attention scores that are significant, this method tackles the problem of quadratic computational overhead present in conventional self-attention mechanisms, thereby lowering the overall computational cost.

### 4.2. Encoder

The encoder can capture long-range dependencies between long sequence inputs. When memory is constrained, the attention distillation mechanism halves the time dimension of features in each layer, enabling the handling of longer input sequences. The INFORMER model utilizes the distillation operation to assign higher weights to dominant attention features, generating concentrated self-attention feature maps in the subsequent layer. The computation from layer *j* to layer j+1 is given by

(6) $$X_{j+1}^{t}=\mathrm{MaxPool}\left(\mathrm{ELU}\left(Conv1d\left({\left[X_{j}^{t}\right]}_{\mathrm{AB}}\right)\right)\right)$$

In the formula, ${\left[X_{j}^{t}\right]}_{AB}$ represents the attention block, while Conv1d denotes the one-dimensional convolution on the time series, followed by max pooling with the ELU activation function. The method of halving the length of the data sequence at each layer reduces memory consumption during the computation process, addressing the high memory usage issue of transformers.

### 4.3. Decoder

The decoder of the INFORMER model is composed of multi-head probabilistic sparse attention layers, position-wise feed-forward networks, dense layers, and complete attention layers. A single forward pass through the decoder predicts the output for long sequences, utilizing a masked attention mechanism for the fully connected layer to produce the final result. The input to the fully connected layer consists of two parts: the historical sequence and the target placeholder sequence. A portion of the historical sequence, close to the predicted target, is dynamically sampled as the “learning value”, while the target placeholder sequence has a length equal to the predicted sequence length, with “0” serving as a placeholder. This setup outputs all predicted values, enhancing the efficiency of long sequence prediction outputs. INFORMER employs batch generative prediction, allowing it to directly output all predicted values as given by

(7) $$\begin{array}{c} X_{\mathrm{feed\_de}}^{t}=\mathrm{Concat}\left(X_{\mathrm{token}\;}^{t},X_{0}^{t}\right)\in R^{\left(L_{\mathrm{token}\;}+L_{y}\right)\times d_{\mathrm{model}\;}}\\ X_{\mathrm{token}\;}^{t}\in R^{\left(L_{\mathrm{token}\;}+L_{y}\right)\times d_{\mathrm{model}\;}} \end{array}$$

In the formula, $X_{\mathrm{feed\_de}}^{t}$ represents the sequence used for learning, and $X_{\mathrm{token}\;}^{t}$ denotes the length of the learning sequence. $X_{\mathrm{token}}^{t}$ represents the historical load sequence used as a reference, while the length of the predicted sequence can be adjusted. $X_{0}^{t}\in R^{L_{y}\times d_{\mathrm{model}\;}}$ is the placeholder for the predicted results, and $X_{0}^{t}$ contains the timestamps of the target sequence.

## 5. DBSCAN-PCA-INFORMER-Based Digital Microfluidic System Droplet Motion Time Prediction Model

### 5.1. Model Structure Design

**Figure 3:**
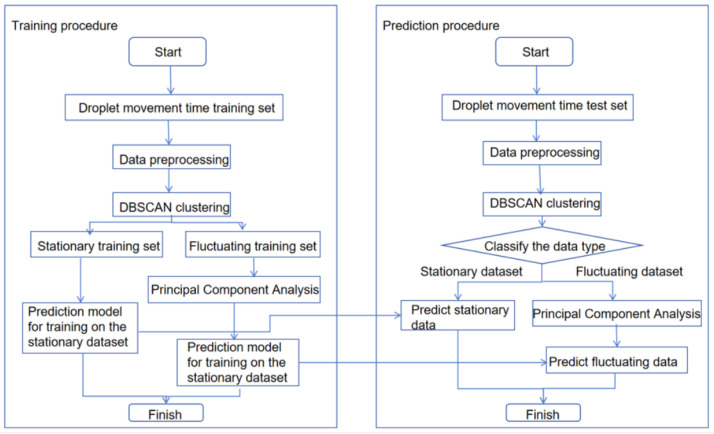
Diagram of droplet movement time prediction model.

The workflow for constructing the prediction model is illustrated in Figure 3, with the goal of developing a droplet motion time prediction model using DBSCAN-PCA-INFORMER for digital microfluidic systems. First, the droplet motion times measured by the machine vision-based digital microfluidic driving control system are preprocessed and used as data samples. The dataset for droplet motion from four chips serves as the training set, while the droplet motion time from one chip is used as the test set. Initially, the training set data is clustered using DBSCAN into a compact steady training set and a dispersed fluctuating training set. For the fluctuating training set, PCA is employed to extract the principal component sequences with high eigenvalues, enabling the identification of similar data for subsequent predictions. The steady training set and the fluctuating dataset are then input into the INFORMER model for training, resulting in corresponding weight files. For the test set, DBSCAN is used again for classification; the steady dataset utilizes the appropriate weight file for prediction, while the fluctuating dataset employs the weight file obtained from training on the PCA-processed fluctuating training set for prediction. Finally, the INFORMER model generates the prediction results, and its performance is assessed through metrics like root mean square error (RMSE) and the coefficient of determination. To verify the model’s generalization ability, test samples measured at varying driving voltages are used, and the model parameters are iteratively refined to develop the final prediction model.

In this study, the DBSCAN-INFORMER model is constructed using the PyTorch framework, with specific model parameter settings detailed in Table 1. First, the optimal parameters for DBSCAN are determined through experimentation. The DBSCAN algorithm is a clustering method that analyzes the number of samples around a central point, guided by two parameters. One is the radius, which affects the range of sample points considered in the calculation. The other is the smallest number of samples needed to constitute a cluster. The first parameter is provided through experimentation. To ensure the sensitivity of the clustering algorithm, the second parameter is set to 0.4.

To assess the clustering results across various radii, the silhouette coefficient serves as the evaluation metric. The formula for calculating the silhouette coefficient is as follows:

(8) $$s=\frac{b-a}{\max(a,\;b)}$$

In the formula, S represents the silhouette coefficient of the sample, and the value of s indicates the quality of the DBSCAN clustering results. The range of s is from −1 to 1, with clustering results falling between these two values. A value of 1 signifies a highly ideal clustering result, where all data points are entirely distinguishable, while −1 represents poor clustering effectiveness, with no data points being distinguishable. b represents the mean distance between the sample and samples in other clusters, while a denotes the mean distance between the sample and samples within the same cluster. The average of the silhouette coefficients for all samples is calculated as the evaluation metric for the DBSCAN results.

**Figure 4:**
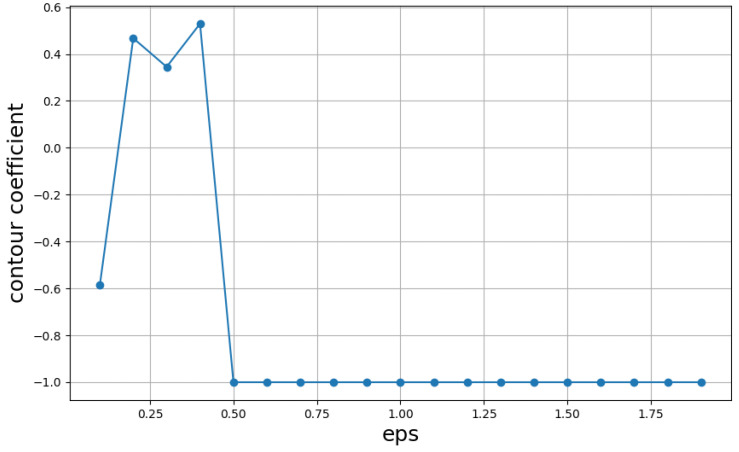
Silhouette coefficients under different parameters.

The clustering results of DBSCAN with different radii are evaluated using the silhouette coefficient formula. The results, shown in Figure 4, indicate that when the radius Eps is set to 0.4, the silhouette coefficient reaches its maximum, suggesting optimal parameters. Subsequently, PCA is applied to the clustered dataset to perform dimensionality reduction on the fluctuating dataset, mitigating the impact of abrupt signals on the feature sequence and enhancing the stability of predictive performance.

The comparison method for the INFORMER model is similar, and the final parameter table is as follows. The dataset, after clustering and dimensionality reduction, is fed into the INFORMER for training. In the INFORMER model, the encoder and decoder process different types of data: the encoder takes a long sequence of historical data, while the decoder handles a short sequence combined with a series of zero values matching the prediction step length, where the zero values act as placeholders for the predicted values. When the data enters the encoder, it passes through multiple processes in the multi-head probabilistic sparse self-attention module, resulting in an intermediate output.The decoder’s input data first undergoes multi-head probabilistic sparse self-attention operations, followed by multi-head self-attention operations utilizing the encoder’s intermediate results. A fully connected layer then adjusts the output dimensions to generate the prediction results. These predictions are refined through backpropagation, continually optimizing the model. After training, the model can accept new droplet motion time data as input and predict future trends.

### 5.2. Evaluation Metrics

Model performance is evaluated using MSE, MAE, MAPE, and $R^{2}$, with their respective calculation formulas as follows:

(9) $$\;\mathrm{MSE}\;=\frac{1}{N}\sum_{i=1}^{N}{\left(y_{i}-{\hat{y}}_{i}\right)}^{2}$$

(10) $$\;\mathrm{MAE}\;=\frac{1}{N}\sum_{i=1}^{N}\left|{\hat{y}}_{i}-y_{i}\right|$$

(11) $$\;\mathrm{MAPE}\;=\frac{1}{N}\sum_{i=1}^{N}\left|\frac{{\hat{y}}_{i}-y_{i}}{y_{i}}\right|$$

(12) $$R^{2}=1-\frac{\sum_{i=1}^{N}{\left(y_{i}-{\hat{y}}_{i}\right)}^{2}}{\sum_{i=1}^{N}{\left(y_{i}-\bar{y}\right)}^{2}}$$

In these formulas, N is the sample size, $y_{i}$ represents the true values, ${\hat{y}}_{i}$ denotes the predicted values from the model, and $\bar{y}$ is the mean of the true values.

## 6. Experiments and Discussion

### 6.1. Device Fabrication and Dataset Collection

**Figure 5:**
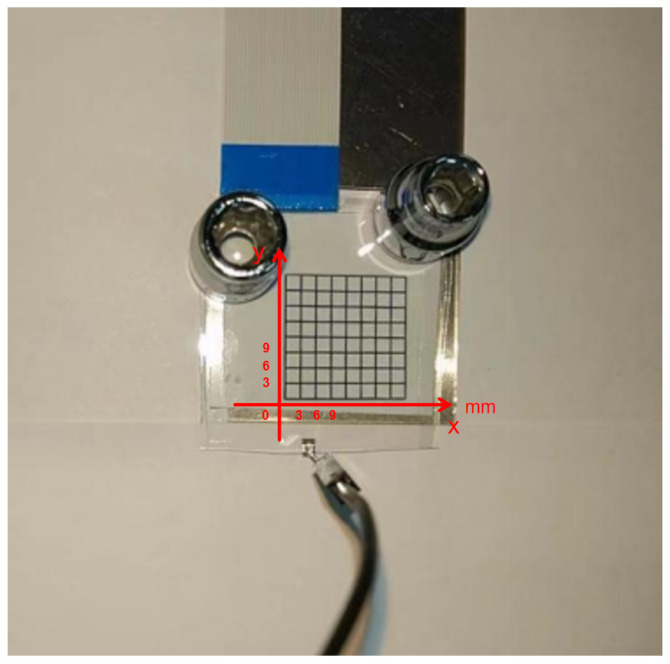
Physical layout of the designed bipolar plate EWOD device used in this experiment.

In this study, we utilized micro-mechanical fabrication techniques to manufacture the digital microfluidic chips required for this research in a clean room environment. The physical layout of the device is shown in Figure 5. To simplify the fabrication process and reduce time, we spin-coated a 1000 nm thick layer of Teflon AF1600 material (Chemours, Wilmington, NC, USA) on the ITO layer surfaces of the upper and lower plates of the device, serving as both the dielectric layer and hydrophobic layer. We used SU-8 photoresist (KAYAKU, Westborough, MA, USA) to create support walls for the upper and lower plates of the EWOD device, ensuring sufficient movement space for the droplets between the two plates. The structural parameters of the EWOD device used in this study are as follows: the driving electrodes are square-shaped with a side length of 3 mm, the distance between the driving electrodes is 25 μm, the gap between the upper and lower plates of the device is approximately 2 mm, and the droplet volume is about 5 μL, with a horizontal diameter of approximately 4 mm on the EWOD device.

**Figure 6:**
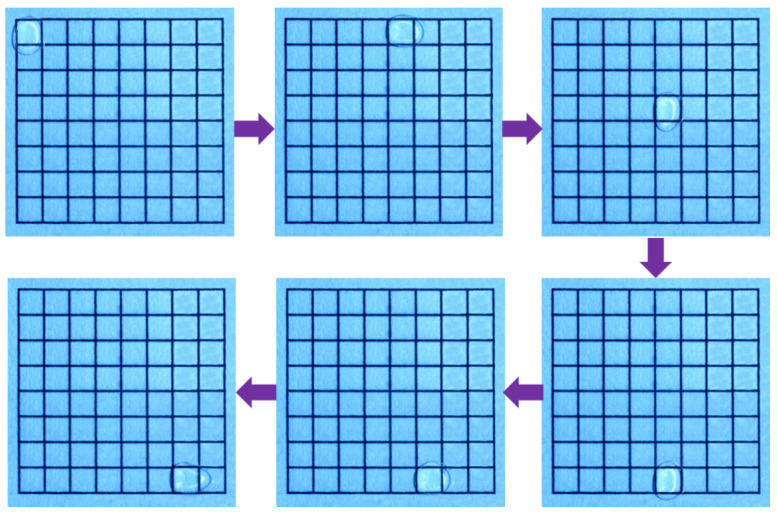
The transport of micro-droplets on the EWOD device from the starting electrode (top-left corner) to the destination electrode (bottom-right corner).

To ensure the stability and reliability of the data while minimizing human intervention, the entire process of driving and timing recording was managed by the machine vision-based digital microfluidic drive control system proposed by the research team [21]. Figure 6 presents a series of screenshots showing the continuous transport of micro-droplets on the EWOD device. To ensure data reliability and continuity, all micro-droplet transport paths in this study were directed from the starting electrode at the top-left corner to the destination electrode at the bottom right corner.

**Figure 7:**
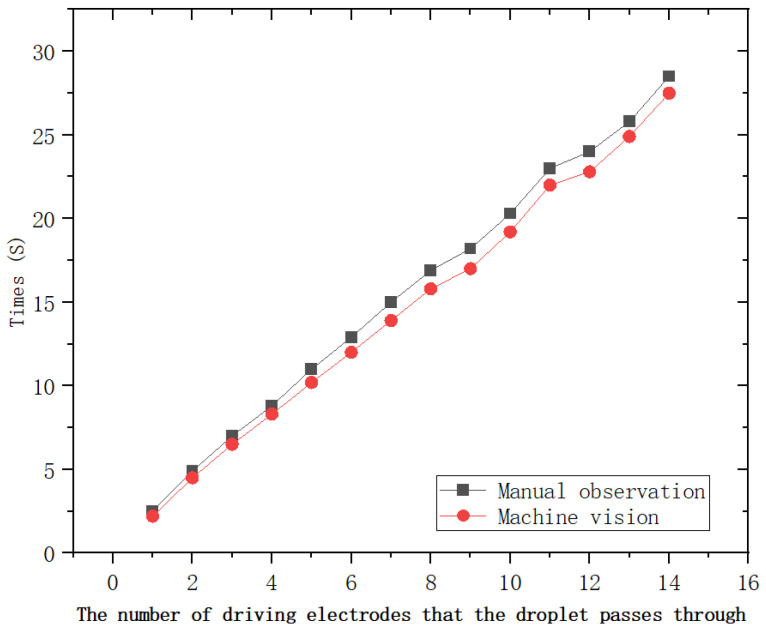
Results of the machine vision-based digital microfluidic drive control system.

In this subsection, we first validate and compare the designed digital microfluidic drive control scheme using manual observation and recording methods. The results of the verification experiments are shown in Figure 7. The experimental results demonstrate that the droplet motion times measured by the machine vision-based digital microfluidic drive control system closely match those obtained through manual observation, with an exceptionally low error rate. Therefore, the droplet motion time data collected by this system can be used for predictions with high reliability.The machine-vision-based digital microfluidic control system operates at a sampling frequency of 30 Hz, and the total number of samples corresponding to the time required for a droplet to fully reach the next electrode is 14,400.

### 6.2. Data Preprocessing

Due to varying device conditions across different chips, even with the same number of experiments, the extent of degradation may differ, thereby affecting the authenticity and accuracy of the model outputs. Therefore, data preprocessing is necessary before prediction. During the time series forecasting phase, data normalization is employed to enhance resilience to outliers, speed up the network’s convergence, and boost the model’s effectiveness and generalization capability. This is detailed in Equation (13).

(13) $$\hat{x}=\frac{x-x_{\min}}{x_{\max}-x_{\min}}$$

In Equation (13), $\hat{x}$ is the normalized value, x represents the droplet motion time data, and $x_{\min}$ and $x_{\max\;}$ are the minimum and maximum values within the droplet motion time data, respectively.

### 6.3. Model Training

The original data were collected at a driving voltage of 80 V, utilizing the droplet motion time parameters from the first four digital microfluidic chips as feature inputs, with the droplet motion time from the fifth chip serving as the data label. This forms a validation dataset designed to assess the training performance of the prediction model.

**Figure 8:**
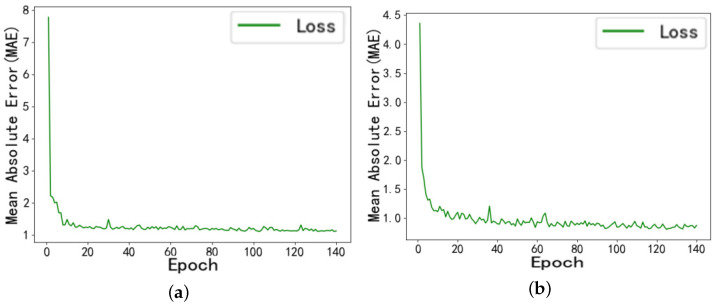
(**a**) Training error graph of the LSTM prediction model. (**b**) Training error graph of the DBSCAN-PCA-INFORMER prediction model.

To validate the feasibility of the droplet motion time prediction method introduced in this study, we compared it with the LSTM prediction model. During the training process, the convergence of training errors for both models is shown in Figure 8.

Under the same training dataset, the DBSCAN-PCA-INFORMER prediction model constructed in this study achieved a MAE of 0.9887 for the training set in the final training cycle. Compared to the LSTM prediction model, its MAE value is reduced by 4.6%, reaching 1.0368. This result effectively verifies that the combined model employed in this study converges quickly and provides high reliability in predicting droplet motion times.

**Figure 9:**
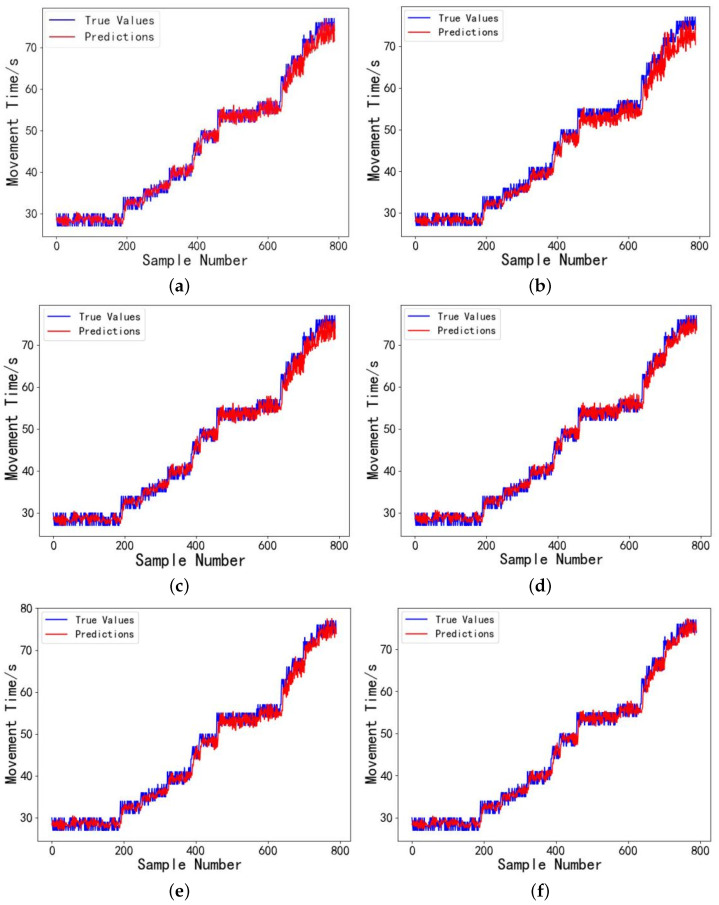
(**a**) Droplet motion time prediction results for the digital microfluidic system using the LSTM model. (**b** Droplet motion time prediction results for the digital microfluidic system using the FEDformer model. (**c**) Droplet motion time prediction results for the digital microfluidic system using the Itransformer model. (**d**) Droplet motion time prediction results for the digital microfluidic system using the INFORMER model. (**e**) Droplet motion time prediction results for the digital microfluidic system Using the DBSCAN-INFORMER model. (**f**) Droplet motion time prediction results for the digital microfluidic system using the DBSCAN-PCA-INFORMER model.

The model’s prediction performance is evaluated using the test set, analyzing how various models predict actual droplet motion times. Figure 9 presents the error comparison between the actual droplet motion times in the test set and the predicted values from LSTM, INFORMER, DBSCAN-INFORMER, and DBSCAN-PCA-INFORMER models. A total of 790 sample data points of droplet motion times were measured at a driving voltage of 80 V. The experimental results demonstrate that at 80 V, the devices experience a significant increase in damage, resulting in greater instability in the droplet motion time data. Additionally, the prediction errors of the LSTM model fluctuate considerably, particularly performing poorly near extreme points. In contrast, the INFORMER model exhibits smaller prediction errors, with the DBSCAN-PCA-INFORMER model showing lower prediction errors across multiple data ranges, especially in complex nonlinear regions where the combined model performs exceptionally well. It is evident that using PCA for dimensionality reduction and extracting the main components from the fluctuating dataset minimizes the interference of irrelevant signals, allowing the INFORMER prediction model to more effectively capture key feature information across different data ranges, resulting in predictions that are closer to the actual values and with higher reliability.

Table 2 presents the performance of six models-LSTM, FEDformer, iTransformer, INFORMER, DBSCAN-INFORMER, and DBSCAN-PCA-INFORMER on the droplet motion time prediction task. The baseline LSTM yields an MSE of 3.8535, MAE of 1.5153, MAPE of 3.3847, and $R^{2}$ of 0.9836. FEDformer reduces the MSE to 3.8234, a reduction of 0.8%, brings the MAE down to 1.4945 (a 1.4% decrease) and lowers the MAPE to 3.3702 (a 0.4% decrease), while $R^{2}$ rises slightly to 0.9838. iTransformer further lowers the MSE to 3.7578, cutting it by 2.5%, reduces the MAE to 1.4721 (a 2.8% decrease) and the MAPE to 3.3407 (a 1.3% decrease), achieving an $R^{2}$ of 0.9841. Replacing the baseline with the standard INFORMER brings the MSE down to 3.7003, a 4.0% reduction, reduces the MAE to 1.4585 (a 3.8% decrease) and the MAPE to 3.3534 (a 1.0% decrease), and boosts $R^{2}$ to 0.9843. Applying DBSCAN filtering before the INFORMER yields an MSE of 3.4103, MAE of 1.4609 and MAPE of 3.3283, with $R^{2}$ rising to 0.9855. Finally, combining DBSCAN and PCA preprocessing achieves the best results: the MSE falls to 3.1925, a reduction of 17.1%; the MAE drops to 1.3661, down by 9.9%; the MAPE decreases to 3.1813, reduced by 6.0%; and $R^{2}$ increases to 0.9864, clearly demonstrating the superiority of the DBSCAN-PCA-INFORMER model.

**Figure 10:**
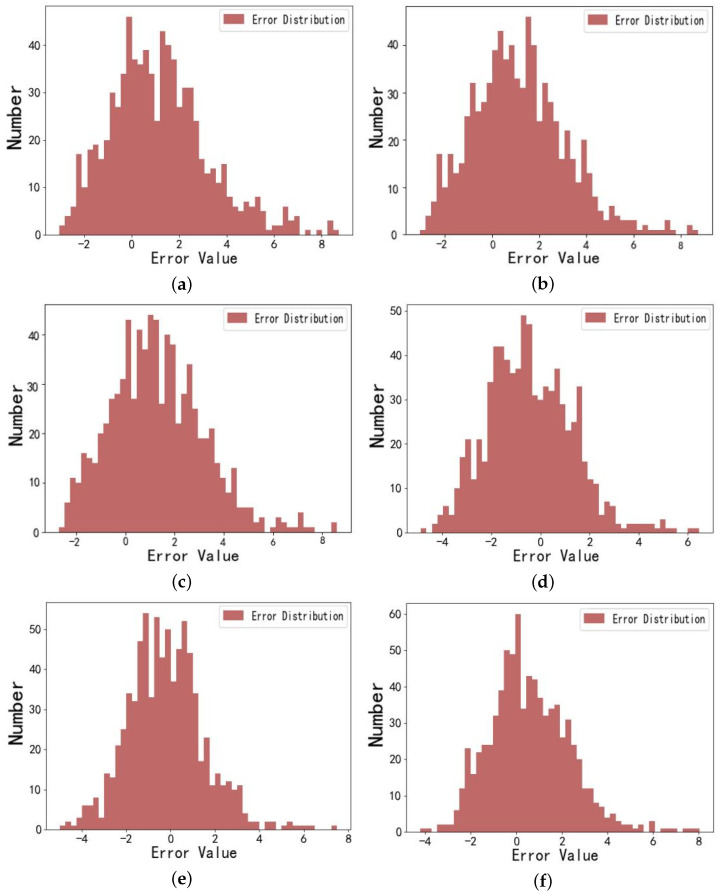
(**a**) Error distribution graph of droplet motion time prediction for the digital microfluidic system using the LSTM model. (**b**) Error distribution graph of droplet motion time prediction for the digital microfluidic system using the FEDformer model. (**c**) Error distribution graph of droplet motion time prediction for the digital microfluidic system using the iTransformer model. (**d**) Error distribution graph of droplet motion time prediction for the digital microfluidic system using the INFORMER model. (**e**) Error distribution graph of droplet motion time prediction for the digital microfluidic system using the DBSCAN-INFORMER model. (**f**) Error distribution graph of droplet motion time prediction for the digital microfluidic system using the DBSCAN-PCA-INFORMER model.

The prediction error distributions for the LSTM, FEDFORMER, iTRANSFORMER, INFORMER, DBSCAN-INFORMER, and DBSCAN-PCA-INFORMER models are shown in Figure 10. The error distribution is centered around the zero-error line, with values closer to zero indicating smaller discrepancies between predicted and actual values, thus reflecting higher prediction accuracy of the models.

The figure shows that, compared to the LSTM prediction model, the error distribution graphs for the INFORMER, FEDFORMER, iTRANSFORMER, DBSCAN-INFORMER, and DBSCAN-PCA-INFORMER models are narrower and more concentrated around zero. These models exhibit smaller prediction errors, even at points with significant data variation, remaining within the expected range. Notably, the error distribution histogram for the DBSCAN-PCA-INFORMER combined model has 60 occurrences in the zero interval (a greater number of occurrences), while having smaller errors compared to the INFORMER, FEDFORMER, iTRANSFORMER and DBSCAN-INFORMER models. This indicates that the DBSCAN-PCA-INFORMER model possesses higher precision and accuracy in predicting droplet motion times, with predictions closer to actual values, thus validating its effectiveness and robustness in improving prediction accuracy.

To further evaluate the practical applicability of the proposed model, we measured the inference time of each model under identical hardware and software conditions. All experiments were conducted on a system equipped with an Intel Core i7-14650 CPU and 16 GB RAM, using PyTorch 1.13.1 without GPU acceleration to simulate a typical real-time deployment environment.

The average inference time for predicting 100 samples is summarized in Table 3. As shown, traditional models such as LSTM and FEDformer require longer inference times due to their sequential computation or complex decomposition structures. In contrast, the proposed DBSCAN-PCA-INFORMER model achieves competitive inference efficiency, owing to its sparse attention mechanism and reduced input dimensionality via PCA.

These results demonstrate that, beyond achieving higher prediction accuracy, the DBSCAN-PCA-INFORMER model also maintains efficient inference speed, making it suitable for integration into real-time microfluidic monitoring systems.

### 6.4. Performance Evaluation of the Model Under Different Driving Voltages

**Figure 11:**
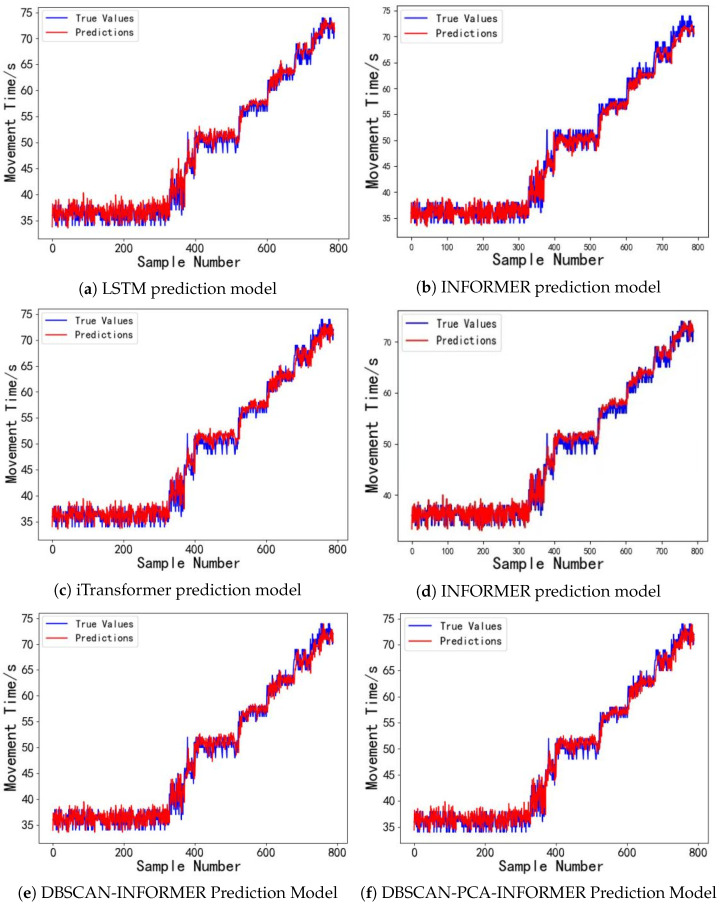
(**a**) Droplet motion time prediction results for the digital microfluidic system based on the LSTM model at 30 V driving voltage. (**b**) Droplet motion time prediction results for the digital microfluidic system based on the FEDformer model at 30 V driving voltage. (**c**) Droplet motion time prediction results for the digital microfluidic system based on the iTransformer model at 30 V driving voltage. (**d**) Droplet motion time prediction results for the digital microfluidic system based on the INFORMER model at 30 V driving voltage. (**e**) Droplet motion time prediction results for the digital microfluidic system based on the DBSCAN-INFORMER model at 30 V driving voltage. (**f**) Droplet motion time prediction results for the digital microfluidic system based on the DBSCAN-PCA-INFORMER model at 30 V driving voltage.

**Figure 12:**
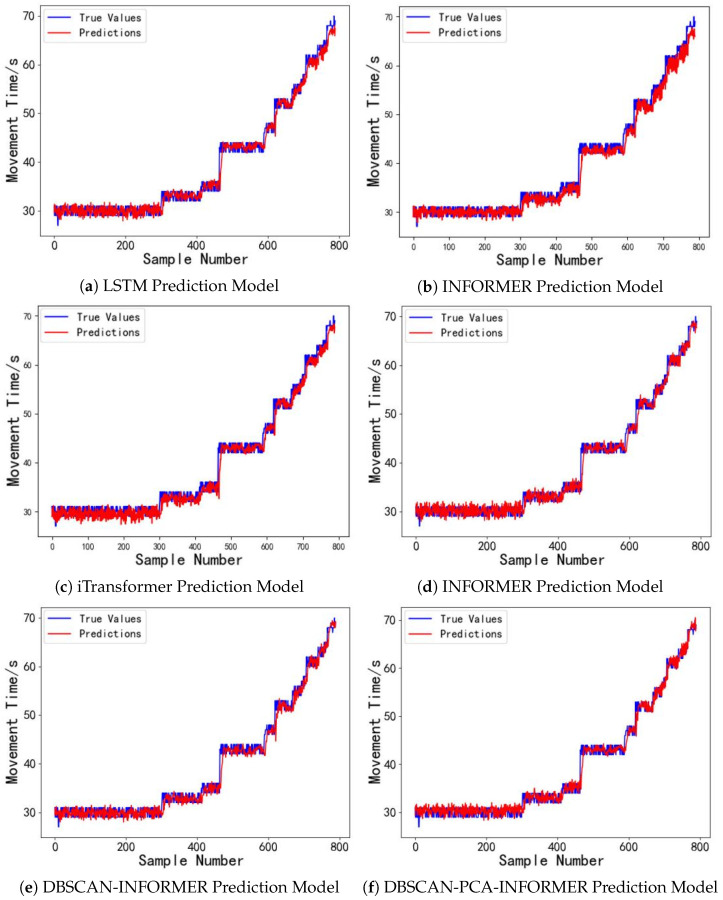
(**a**) Droplet motion time prediction results for the digital microfluidic system based on the LSTM Model at 50 V driving voltage. (**b**) Droplet motion time prediction results for the digital microfluidic system based on the FEDformer Model at 50 V driving voltage. (**c**) Droplet motion time prediction results for the digital microfluidic system based on the iTransformer model at 50 V driving voltage. (**d**) Droplet motion time prediction results for the digital microfluidic system based on the INFORMER model at 50 V driving voltage. (**e**) Droplet motion time prediction results for the digital microfluidic system based on the DBSCAN-INFORMER model at 50 V driving voltage. (**f**) Droplet motion time prediction results for the digital microfluidic system based on the DBSCAN-PCA-INFORMER model at 50 V driving voltage.

With a driving voltage of 80 V, the model introduced in this study exhibits outstanding performance in predicting the droplet motion time for digital microfluidic chips. We will now examine the model’s predictive performance at driving voltages of 30 V and 50 V. The dataset includes 3200 sample data points of droplet motion times from the first four digital microfluidic chips for training, while 800 sample data points from the fifth chip are utilized as the evaluation set. The forecasting outcomes are illustrated in Figure 11 and Figure 12. The experimental results in Figure 11 indicate that under long time series inputs, the LSTM prediction model faces a gradual loss of temporal information within the predicted time interval (600, 800), leading to a decline in model performance and substantial deviations between the predicted and actual droplet motion time values. This is due to the long-term voltage drive causing damage to the devices, resulting in significant uncertainty in the droplet motion times. In contrast, the INFORMER and DBSCAN-INFORMER models effectively capture the trend of data fluctuations, minimizing the discrepancy between predicted and actual values. Furthermore, the DBSCAN-PCA-INFORMER prediction model minimizes the error, showcasing its remarkable advantage in accurately capturing sudden signal changes. As shown in Figure 12, within the predicted time interval (700, 800), the DBSCAN-PCA-INFORMER model provides droplet motion time predictions that are closest to the actual values compared to the other models, with both exhibiting nearly identical trends, thus demonstrating higher reliability.

Figure 11 and Figure 12 and Table 4 demonstrate that the DBSCAN-PCA-INFORMER prediction model accurately predicts droplet motion times at different driving voltages of 30 V and 50 V, achieving R^2^ values greater than 0.98. The MSE, MAE, and MAPE metrics all outperform those of the LSTM, FEDformer, iTransformer, INFORMER, and DBSCAN-INFORMER prediction models. In other words, the DBSCAN-PCA-INFORMER prediction model demonstrates better robustness than the other models. This is primarily attributed to its integration of the DBSCAN clustering algorithm and PCA, which enables more effective feature extraction and dimensionality reduction, thereby enhancing predictive accuracy. Therefore, the proposed prediction model exhibits strong generalization capability in predicting droplet motion times under different driving voltages.

## 7. Conclusions

This paper proposes a DBSCAN-PCA-INFORMER-based prediction model for predicting droplet motion times in digital microfluidic systems. The model first utilizes droplet motion time data obtained from a machine vision-based digital microfluidic driving control system for clustering and dimensionality reduction, effectively enhancing the stability of prediction performance. Subsequently, the processed dataset is input into the INFORMER model, which performs multiple operations through a multi-head probabilistic sparse self-attention module to learn key temporal information about the characteristics of each chip. The decoder then performs multi-head probabilistic sparse self-attention operations based on the intermediate results provided by the encoder. Finally, a fully connected layer integrates the learned information to predict the output of droplet motion time. This study compares and analyzes the prediction performance of LSTM, FEDformer, iTransformer, INFORMER, DBSCAN-INFORMER, and DBSCAN-PCA-INFORMER models. The results show that clustering the dataset using DBSCAN and extracting the main components of the fluctuating dataset through PCA enables the DBSCAN-PCA-INFORMER model to have a stronger capability to capture important information from various time series, facilitating the identification of highly similar series for predictions, thereby improving prediction accuracy. The INFORMER model, which incorporates a multi-head self-attention mechanism, demonstrates significant advantages over traditional models like LSTM, as it better captures the trends and seasonality of droplet motion times while considering the damage conditions of digital microfluidic chips under different driving voltages, further enhancing prediction accuracy. By removing abnormal segments using DBSCAN and preserving major variance directions through PCA, this method effectively eliminates high-frequency noise while retaining key information. The model can automatically attenuate information from droplet “sticking” regions caused by electrode aging, implicitly extracting fault indicators from attention weights, enabling seamless integration with subsequent path planning algorithms.

Additionally, the model’s performance under different driving voltages is verified through generalization and robustness simulations. A predictive analysis is conducted comparing the LSTM, FEDformer, iTransformer, INFORMER, DBSCAN-INFORMER, PCA-INFORMER, and DBSCAN-PCA-INFORMER models, with the DBSCAN-PCA-INFORMER model exhibiting superior predictive performance. At a driving voltage of 30 V, the DBSCAN-PCA-INFORMER model achieved an $R^{2}$ of 0.9806, with an MSE of 3.0649, an MAE of 1.3312, and a MAPE of 2.9962. At a driving voltage of 50 V, the model achieved an $R^{2}$ of 0.9837, with an MSE of 2.1541, an MAE of 1.1072, and a MAPE of 2.9393. These results indicate that the model maintains high prediction accuracy even under different driving voltages, demonstrating good generalization capability and robustness. The organic integration of DBSCAN and PCA preprocessing with the INFORMER model allows for the extraction of soft fault indicators from sparse attention weights, enabling rapid identification of underperforming driving electrodes without requiring any hardware modifications. This study, by introducing a pipeline that integrates DBSCAN-PCA preprocessing with INFORMER prediction, fills the prior research gap of not using droplet motion time predictions to detect driving electrode faults and guide subsequent path planning.

Through the proposed DBSCAN-PCA-INFORMER model for predicting droplet motion times in digital microfluidic systems, we can infer the status of digital microfluidic devices and the degree of fault in the driving electrodes. This provides strong support for the scientific assessment of device health, aiming to minimize the impact of driving electrode faults on various droplet applications and providing a solid foundation for research and applications in related fields.

## Figures and Tables

**Table 1:** Model parameter table.

Model	Name	Parameter
DBSCAN	Eps	0.4
	Min_samples	5
INFORMER	BatchSize	64
	Learning Rate	0.0005
	Encode Input Size	7
	Decode Input Size	7
	Encoder Layer	2
	Decoder Layer	1
	Epoch	140
	Loss	MeanSquareError
	Optimizer	Adam Optimizer

**Table 2:** Performance Comparison of LSTM, FEDFORMER, iTRANSFORMER, INFORMER, DBSCAN-INFORMER, and DBSCAN-PCA-INFORMER Prediction Models.

Model	MSE	MAE	MAPE	R^2^
LSTM	3.8535	1.5153	3.3847	0.9836
FEDFORMER	3.8234	1.4945	3.3702	0.9838
ITRANSFORMER	3.7578	1.4721	3.3407	0.9841
INFORMER	3.7003	1.4585	3.3534	0.9843
DBSCAN-INFORMER	3.4103	1.4609	3.3283	0.9855
DBSCAN-PCA-INFORMER	3.1925	1.3661	3.1813	0.9864

**Table 3:** Inference time comparison of LSTM, INFORMER, FEDFORMER, iTRANSFORMER, DBSCAN-INFORMER, and DBSCAN-PCA-INFORMER models (per 100 samples).

Model	Inference Time per 100 Samples (ms)
LSTM	92.4
FEDFORMER	101.7
ITRANSFORMER	89.3
INFORMER	68.9
DBSCAN-INFORMER	66.5
DBSCAN-PCA-INFORMER	59.1

**Table 4:** Comparison of droplet motion time prediction performance under different driving voltages.

Driving Voltage/V	Model	MSE	MAE	MAPE	R^2^
30	LSTM	3.7343	1.4826	3.3351	0.9763
	FEDFORMER	3.5035	1.4550	3.2890	0.9778
	ITRANSFORMER	3.4070	1.4210	3.1520	0.9784
	INFORMER	3.4873	1.4402	3.1565	0.9779
	DBSCAN-INFORMER	3.1888	1.3737	3.1472	0.9798
	DBSCAN-PCA-INFORMER	3.0649	1.3312	2.9962	0.9806
50	LSTM	2.8270	1.2903	3.4057	0.9786
	FEDFORMER	2.8105	1.2872	3.3921	0.9787
	ITRANSFORMER	2.7708	1.2834	3.3407	0.9790
	INFORMER	2.7511	1.2802	3.3248	0.9792
	DBSCAN-INFORMER	2.5846	1.2438	3.3219	0.9805
	DBSCAN-PCA-INFORMER	2.1541	1.1072	2.9393	0.9837

## Data Availability

The original contributions presented in this study are included in the article. Further inquiries can be directed to the corresponding authors.
